# Molecular characterization of immunogenic cell death indicates prognosis and tumor microenvironment infiltration in osteosarcoma

**DOI:** 10.3389/fimmu.2022.1071636

**Published:** 2022-12-09

**Authors:** Zhongyue Liu, Binfeng Liu, Chengyao Feng, Chenbei Li, Hua Wang, Haixia Zhang, Ping Liu, Zhihong Li, Shasha He, Chao Tu

**Affiliations:** ^1^Department of Orthopaedics, The Second Xiangya Hospital of Central South University, Changsha, Hunan, China; ^2^Hunan Key Laboratory of Tumor Models and Individualized Medicine, The Second Xiangya Hospital of Central South University, Changsha, Hunan, China; ^3^Department of Oncology, The Second Xiangya Hospital of Central South University, Changsha, Hunan, China

**Keywords:** immunogenic cell death, osteosarcoma, molecular characteristics, tumor microenvironment infiltration, prognosis

## Abstract

**Introduction:**

Osteosarcoma (OS) is a highly aggressive bone malignancy with a poor prognosis, mainly in children and adolescents. Immunogenic cell death (ICD) is classified as a type of programmed cell death associated with the tumor immune microenvironment, prognosis, and immunotherapy. However, the feature of the ICD molecular subtype and the related tumor microenvironment (TME) and immune cell infiltration has not been carefully investigated in OS.

**Methods:**

The ICD-related genes were extracted from previous studies, and the RNA expression profiles and corresponding data of OS were downloaded from The Cancer Genome Atlas and Gene Expression Omnibus database. The ICD-related molecular subtypes were classed by the "ConsensusclusterPlus" package and the construction of ICD-related signatures through univariate regression analysis. ROC curves, independent analysis, and internal validation were used to evaluate signature performance. Moreover, a series of bioinformatic analyses were used for Immunotherapy efficacy, tumor immune microenvironments, and chemotherapeutic drug sensitivity between the high- and low-risk groups.

**Results:**

Herein, we identified two ICD-related subtypes and found significant heterogeneity in clinical prognosis, TME, and immune response signaling among distinct ICD subtypes. Subsequently, a novel ICD-related prognostic signature was developed to determine its predictive performance in OS. Also, a highly accurate nomogram was then constructed to improve the clinical applicability of the novel ICD-related signature. Furthermore, we observed significant correlations between ICD risk score and TME, immunotherapy response, and chemotherapeutic drug sensitivity. Notably, the in vitro experiments further verified that high GALNT14 expression is closely associated with poor prognosis and malignant progress of OS.

**Discussion:**

Hence, we identified and validated that the novel ICD-related signature could serve as a promising biomarker for the OS's prognosis, chemotherapy, and immunotherapy response prediction, providing guidance for personalized and accurate immunotherapy strategies for OS.

## Introduction

As a primary malignant bone tumor, osteosarcoma (OS) mainly affects children and adolescents, with an approximate annual prevalence of (3-4)/1,000,000 worldwide and a slightly higher incidence in men than in that in women (1, 2). OS was characterized by high invasiveness and early metastasis, with about 10%-25% of them with pulmonary metastasis (3, 4). With the extensive application of neoadjuvant chemotherapy, comprehensive treatment consisting of surgical resection and multi-scheme chemotherapy has become the current standard treatment for almost all patients with OS, significantly improving the overall survival of OS (5). Five-year survival rates for localized OS patients have reached 60-70% (6). However, the prognosis of metastases individual is only 20-30% (7). Moreover, the molecular mechanisms and therapeutic targets are challenging to determine because of the high complexity and heterogeneity between different OS tissue types (8). Recently, accumulating research has revealed that characteristic molecular classifications of OS are potentially effective for the personalized treatment and prognosis prediction of OS. For instance, Yang et al. divided OS patients into two immune subtypes and revealed a novel risk model for the prognosis prediction of OS (9). Therefore, a new molecular subtype is considered a promising approach for the prognosis improvement of OS.

**Figure 1:**
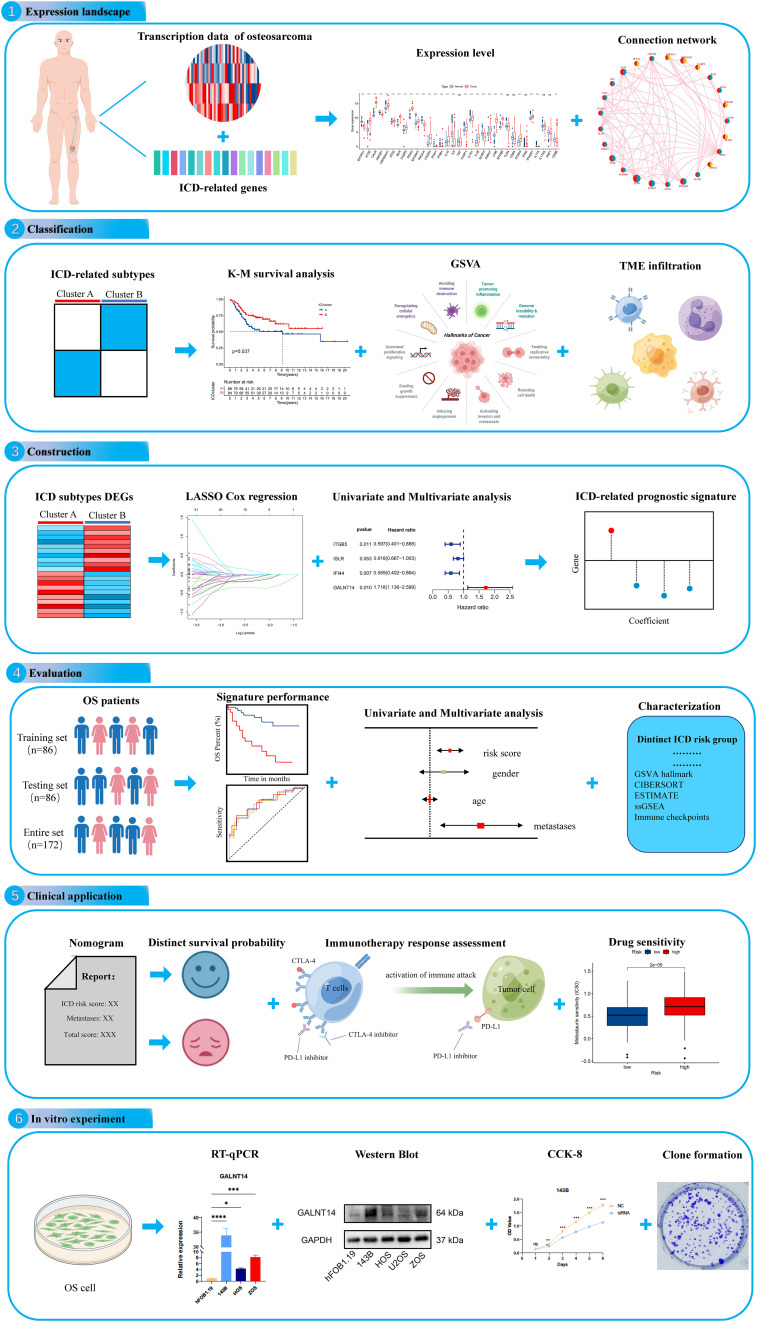
The flow diagram of the present study. ns, no significance, *p < 0.05, **p < 0.01, ***p < 0.001,****p < 0.0001.

Immunogenic cell death (ICD) is a distinctive death form of tumor cells proposed by Casares et al. in 2005. It was characterized by a transformation of cells from non-immunogenic to immunogenic and stimulated tumor immune effects in the body, then resulting in cell death (10). Dying tumor cells release damage-related molecular patterns (DAMPs) when ICD occurs, activating and recruiting antigen-presenting cells and then activating T cells to generate an adaptive immune response against tumor antigens (11, 12). Increasing evidence indicates that ICD is to be a particularly effective strategy for tumors resistant to traditional treatment regimens. As an example, it was shown that the subtype based on ICD could predict prognosis in head and neck squamous cell carcinoma (HNSCC) and response to immunotherapy (13). However, the association of ICD-related genes with the clinical prognosis and anticancer mechanisms of OS is unknown. Therefore, a comprehensive understanding of the molecular characteristics of ICD-related genes may provide insight clues to the cause of OS heterogeneity.In the present study, we tried to investigate the expression profile of ICD-related genes and construct an ICD-associated subtype that could help predict clinical prognosis, immune landscape, and immunotherapy response in OS. The flowchart of our study is presented in **Figure 1**. Initially, we explored the expression landscape of ICD-related genes in OS to preliminary reveals their association with OS. Then, we stratified OS patients into two ICD-related molecular clusters based on ICD-related genes and explored the difference between the two classifications. Next, to understand the role of ICD subtypes in the prognostic assessment of OS patients, we establish a novel ICD-related signature. Also, we performed validation analysis to further validate the predictive performance of the ICD-related signature. To assess the clinical applicability of the novel ICD-related signature, we construct a nomogram, immunotherapy response, and chemotherapeutic drug sensitivity analysis. More importantly, *in vitro* experiments were performed to verify the analytical results. These results are helpful for the prognosis prediction of OS and apply more individualized and effective anticancer treatment strategies for OS patients.

## Materials and methods

### Dataset preprocessing

The related data of RNA sequencing, as well as clinical information, were extracted from The Therapeutically Applicable Research to Generate Effective Treatments database (TARGET, https://ocg.cancer.gov/programs/target) (14) and the Gene Expression Omnibus (GEO, https://www.ncbi.nlm.nih.gov/geo/) (15). In addition, the relevant datasets of normal tissue were downloaded from Genotype-Tissue Expression Project (GTEx, https://www.gtexportal.org/home) database. Patients with incomplete or illegible clinical features were excluded from further analysis. 88 OS and 396 normal tissues were used to explore the expression level of ICD-related genes. A total of 172 OS patients with survival information were obtained from two GEO cohorts (GSE16091 and GSE21257) and TARGET cohorts. To normalize and eliminate batch effects across different cohorts, we initially converted the value of Fragments Per Kilobase per Million (FPKM) with each transcript to millions per kilobase (TPM). Subsequently, a meta-cohort of these four OS cohorts was generated through the “Combat” algorithm. The detailed features of the OS patients are shown in **Table S1**.

### Consensus clustering

The ICD-related genes were retrieved from previous studies for further analysis (16). Next, consensus clustering was performed *via* the “ConsensusclusterPlus” package in R Studio to classify ICD-related molecular subtypes based on these genes. To obtain stable results, the values of clusters (k) varied from two to ten, and the number of iterations was 1000. After that, a gene set variation analysis (GSVA) was carried out to analyze the biological processes among these distinct ICD-related molecular subtypes. The relationship between the clinical feature of OS and ICD-related molecular subtypes was examined using survival analysis, Principal component analysis (PCA) analysis, and heatmap.

### Relationship of molecular classification with the immune status

Next, a series of bioinformatics algorithms were used to explore the immune status among different ICD-related subtypes. First, the ESTIMATE was performed to evaluate the immune, stromal, and ESTIMATE scores of each OS patient and compared the score between these ICD-related classifications (17). The tumor microenvironment (TME) is composed of immune, vascular, extracellular matrix and stromal components and plays a pivotal role during tumor progression and therapy (18). To investigate the TME in OS Cohort, an algorithm, “single-sample gene set enrichment analysis” (ssGSEA), was applied to examine the immune infiltration cells (19). The relationship between the expression levels of PD-1 and PD-L1, as well as the corresponding ICD-related subtypes, were finally analyzed.

### Derivation and verification of the ICD-related risk signature

To quantify the ICD pattern of each OS patient, an ICD-related risk signature was established. Initially, 172 OS patients were divided into training set testing sets with a one-to-one ratio by “caret” package in R software. The training set was used to build the model, while the testing set was used to validate it. After that, the univariate Cox analysis of ICD subtype-related differentially expressed genes (DEGs) was applied to filter genes related to the prognosis of OS patients. Moreover, the least absolute shrinkage and selection operator (LASSO) Cox regression algorithm was carried out to screen prognostic variables. Finally, we selected candidate genes *via* multivariate Cox analysis to construct ICD-related risk signatures. The ICD risk score was reckoned according to this formula: ICD risk score =Σ (coef (i) *Exp (i)). Specifically, coef (i) and Exp (i) are the correlative coefficient and the expression of each gene, respectively. The patients of the training set, testing set, and entire sets, were divided into high and low-risk groups according to the median value of the ICD risk score in the training cohort for subsequent analysis.

### Prognostic and independent analysis

The Kaplan-Meier (K-M) analysis, survival status, and risk curve were conducted to compare the overall survival between the low-risk and high-risk ICD subgroups, respectively. The R package “ggplot2” was used for PCA analysis. Next, the receiver operating characteristic (ROC) curves were generated to estimate the forecasting efficiency of the novel ICD-related risk signature. In addition, the K-M analysis of the clinicopathologic subgroups was utilized to inspect the signature’s stability further. Finally, the univariate and multivariate Cox analysis was conducted to identify whether the ICD risk score is an independent prognosis for OS or not.

### Nomogram and calibration

Nomogram is widely used in the prognosis prediction of cancer and is mainly because the number of statistical models can be reduced to a single numerical evaluation that matches the patient’s individual profile (20). To further verify the predictive values of ICD-related risk scores signature stratified by clinicopathological parameters, we have developed a nomogram to predict the overall survival by using the R package “rms.” At the same time, the predictive accuracy and consistency of nomograms were evaluated by the calibration and ROC curves.

### GSVA

Furthermore, we identify the potential molecular mechanisms between the different risk subgroups. GSVA is a non-parametric and unsupervised method commonly used to assess pathway variation and biological process activity (21). It has transformed gene expression data, which could be used to quantify gene enrichment results and facilitates subsequent statistical analysis. The GSVA was conducted with the R package “GSVA”, and the results were further subjected to differential analysis by the limma package (21, 22). Moreover, significant differential pathways with |logFC|>0.15 and adjusted P-values <0.01 were visualized by a clustered heatmap.

### Immunotherapy response and drug susceptibility analysis

Immunotherapy response and drug susceptibility analysis were carried out to investigate differences in the immunotherapy and therapeutic effects of anticancer agents in OS patients between the two ICD risk groups. Initially, the subclass mapping (submap) was used to predict immunotherapy response differences (23). Similarly, with the R package “pRRophetic”, we figured semi-inhibitory concentration (IC50) values of chemotherapeutic agents and compared the variable IC50 among different risk groups, which could help predict the potential chemotherapeutic agents for OS (24).

### Cell culture

The human normal osteoblast cell line (Human fetal osteoblasts, hFOB1.19) was purchased from American Type Culture Collection (ATCC) and cultured in DMEM/F12 medium (Gibco, United States). Human OS cell lines 143B, and HOS were purchased from ATCC and cultured in MEM (Gibco, United States). Human OS cell line ZOS was gifted by Prof. Kang Tiebang (Sun Yat-Sen University, China) and cultured in DMEM (Gibco, United States). All the cell lines were cultured in 10% fetal bovine serum (Gibco, United States) and 1% antibiotics (penicillin and streptomycin) (NCM Biotech, China). The hFOB1.19 cells were cultured at 34°C, while the rest of the OS cell lines were cultured at 37°C.

### RT-qPCR

Total RNA was extracted from cells *via* RNA Express Total RNA Kit (M050, NCM Biotech, China), and the reverse transcription of RNA was conducted by the Revert Aid First Strand cDNA Synthesis Kit (K1622, Thermo Scientific, United States). Following, the RT-qPCR was executed by Hieff qPCR SYBR Green Master Mix (High Rox Plus) (11203ES, YEASEN Biotech Co., Ltd, China). The 2^-ΔΔCT^ method was selected to evaluate the expressions of the included genes. The primer sequences are presented in **Table S2**.

### Western blot

Total protein was harvested by RIPA buffer (WB3100, NCM, China), and the BCA Protein Quantification Kit (E112-01, Vazyme, China) was used to test and adjust the protein concentrations. Next, proteins (30ug) were separated *via* 10% SDS-PAGE and transferred onto the PVDF membrane. After blocking, incubated the membrane with primary antibodies GAPDH (T0004, Affinity, China), GALNT14 (16939-1-AP, Proteintech, China) overnight at 4°C and with corresponding secondary antibodies anti-Mouse IgG (511103, Zen Bioscience, China), anti-rabbit IgG (7074, CST, USA) for one hour. Finally, the protein bands of the membranes were detected by chemiluminescence with ECL (BIO-RAD, USA). The WB bands were quantitated *via* the software ImageJ.

### Cell transfection

Small interfering RNAs (siRNAs) were purchased from HANBIO (Shanghai, China). The sequences of siRNA-GALNT14 and siRNA-NC are listed in **Table S3**. According to the manual, siRNAs were transfected into cells using LipofectamineTM RNAiMAX (13778-150, Invitrogen, Carlsbad, USA) when 143B cells had 30-50% confluence. After transfection, the efficiency of transfection was detected by RT-qPCR and WB.

### Cell proliferation assay

143B cells were seeded into 96-well plates overnight and cultured continually after being transfected with the GALNT14-siRNA and the corresponding normal control (NC). From the first to the 6^th^ day after transfection, added CCK-8 solution and incubated at 37°C for one hour, followed by the detection of the absorbance values at 450 nm.

### Colony formation assay

After siRNA transfection, 143B cells were seeded into 6-well plates (300 cells/well) and cultured for ten days to form colonies. After that, the cell colonies were washed with PBS and fixed in 4% paraformaldehyde for 30 minutes. Subsequently, the cell colonies were washed three times and followed by staining with 1% crystal violet for 10 minutes. Finally, the number of the stainer cell colonies was observed and recorded under a microscope. And the software ImageJ was used for the counting of colony formation.

### Statistical analysis

The statistical analysis was performed with The R software (version 4.1.0) in this study. The Wilcoxon test was used to compare the difference between the two groups, while the Kruskal-Wallis and one-way ANOVA tests were conducted for differential analysis among the three groups. The Spearman analysis was carried out to investigate the relationship between two variables. Generally, p < 0.05 was considered statistically significant.

## Results

### The expression landscape of ICD-related genes

**Figure 2:**
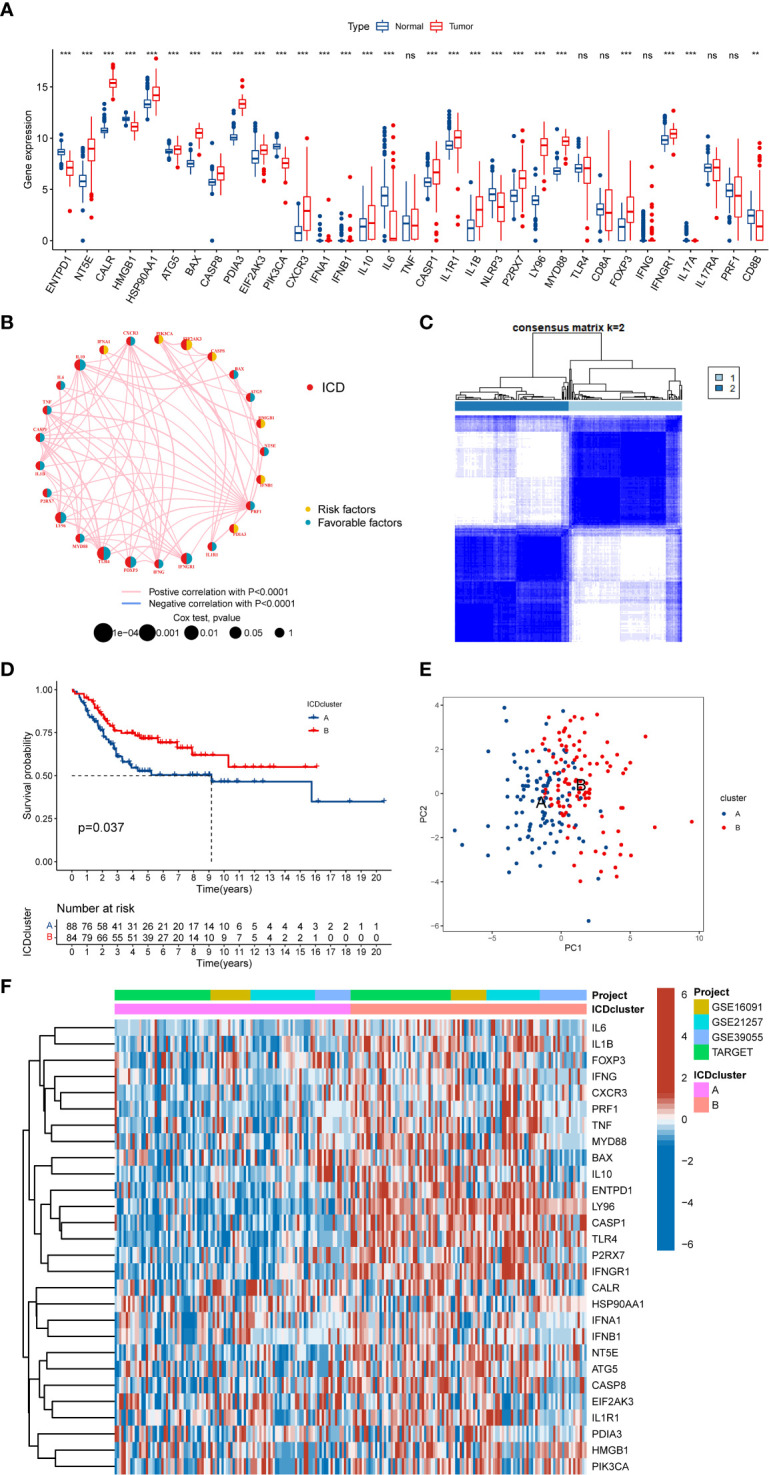
The expression landscape of ICD-related genes in OS. **(A)** The expression of ICD-related genes between OS and normal tissues. **(B)** Interaction among ICD-related genes in OS. The line connecting the ICD-related genes represents their interaction, the red line represents positive correlation, the blue line represents negative correlation, and the line thickness represents the strength of the association between ICD-related genes. The circle size indicates the P-value, the yellow ring represents risk factors, and the blue ring represents favourable factors. **(C)** K = 2 was identified as the optimal value for consensus clustering. **(D)** K-M Survival analysis of two ICD-related subtypes in OS patients. **(E)** PCA analysis indicates a remarkable difference in distinct ICD-related subtypes. **(F)** Differences in ICD-related gene expression levels between the two distinct subtypes. ICD, Immunogenic cell death; OS, Osteosarcoma; K-M, Kaplan-Meier; PCA, Principal component analysis. ns, no significance, **p < 0.01, ***p < 0.001.

We discovered that most of the ICD-related genes were elevated in the OS cohort (**Figure 2A**). For instance, NT5E, CALR, and PDIA3 were upregulated, while IL6, ENTPD1, and NLRP3 were downregulated. Also, several ICD-related genes showed no significance between the OS and normal tissue, such as IL17A and PRF1. Moreover, the univariate Cox regression and Kaplan–Meier (K-M) analysis results display the prognostic role of ICD-related genes (**Table S4**). Next, three ICD-related genes (EIF2AK3, TLR4, and FOXP3) were identified as an independent factor for OS by multivariate Cox regression analysis (**Table 1** and **Figure S1**). Among them, EIF2AK3 is a prognostic risk factor for OS, which is negatively related to the survival rate, while TLR4 and FOXP3 are prognostic protective factors for OS and are positively associated with the survival rate. Furthermore, a comprehensive assessment of ICD-related gene interactions, regulator connections, and prognostic value in OS was presented in the ICD network (**Figure 2B**).

**Table 1:** Multivariate Cox regression analysis of three ICD-related genes associated with OS patients’ prognosis.

Gene	HR	95% CI	P-value
EIF2AK3	1.388730003	0.980576-1.966773	0.064387
TLR4	0.556156675	0.36476-0.847984	0.06408
FOXP3	0.475573337	0.236768-0.955239	0.036739

### Identification of ICD-related classification in OS

To further explore the expression characteristics of ICD-related genes in OS, this OS cohort was subtyped in accordance with the consensus clustering algorithm. The result suggested k = 2 as the optimal choice, and 172 OS patients were divided into two ICD-related classifications: A subtype(n=88) and B subtype(n=84) (**Figure 2C** and **Figure S2**). At the same time, PCA analysis revealed that these two ICD-related subtypes were significantly different, which proved the reliability of the typing (**Figure 2E**). The K-M survival analysis also demonstrated an obvious variance in survival prognosis between the two subtypes. The OS patients in subtype B had a better survival rate than that in subtype A (**Figure 2D**). Furthermore, the heatmap indicated that most ICD-related genes were overexpressed in the B subtype compared to the A subtype (**Figure 2F**). Consequently, the above results showed that the ICD-related subtypes are successfully identified, and the two ICD-related subtypes have significant differences.

### TME features in the ICD-related subtypes

**Figure 3:**
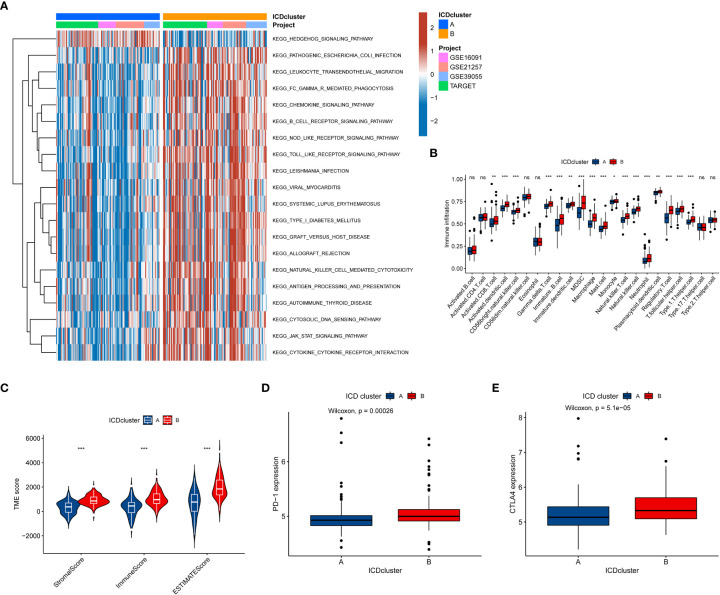
The relationship between the tumor immune cell microenvironments and ICD-related subtypes. **(A)** The GSVA revealed the activation status of biological pathways in two ICD-related molecular subtypes. Red: activation of biological pathways, blue: inhibition of biological pathways. **(B)** The abundance difference of infiltrating immune cell types in the two distinct subtypes. **(C, D)** The levels of expressed PD-1 and CTLA4 in two ICD-related subtypes. (e) The correlation between the TME score and the ICD-related subtype. ICD, Immunogenic cell death, GSVA, Gene set variation analysis, TME, tumor microenvironment. ns, no significance,*p < 0.05, **p < 0.01, ***p < 0.001.

To further explore whether the difference also exists in the signaling pathway between the two different ICD-related subtypes, we performed the GSVA. The results demonstrated that Cluster B was a greatly enriched pathway associated with immune function (**Figure 3A**), such as chemokine, B cell receptor, and NOD-like receptor. To further understand the role of ICD-related genes in the TME, we utilized ssGSEA to compare the enrichment scores of immune cells between two different subtypes. We observed significant differences in the infiltration of most immune cells between the two ICD-related subtypes (**Figure 3B**). The B subtype showed a higher infiltration level than that in subtype A in almost all immune cells, including activated CD8 T cells, activated dendritic cells etc. However, the difference in the infiltration abundance of activated B cell, activated CD4 cell, CD56dim natural killer cell, eosinophil, type 17 T helper cell, and type 2 T helper cell between the two classifications was insignificant. Equally, the ESTIMATE results demonstrated higher TME scores in subtype B, including stromal, immune, and ESTIMATE scores (**Figure 3C**). For immune checkpoints, the expression of two crucial immune checkpoints (PD1 and CTLA4) in subtype B is significantly higher than that in subtype A (**Figures 3D, E**). Generally, these results revealed a close association between the ICD-related subtype and TME.

### Comprehensive analysis of DEGs associated with ICD-related phenotype

**Figure 4:**
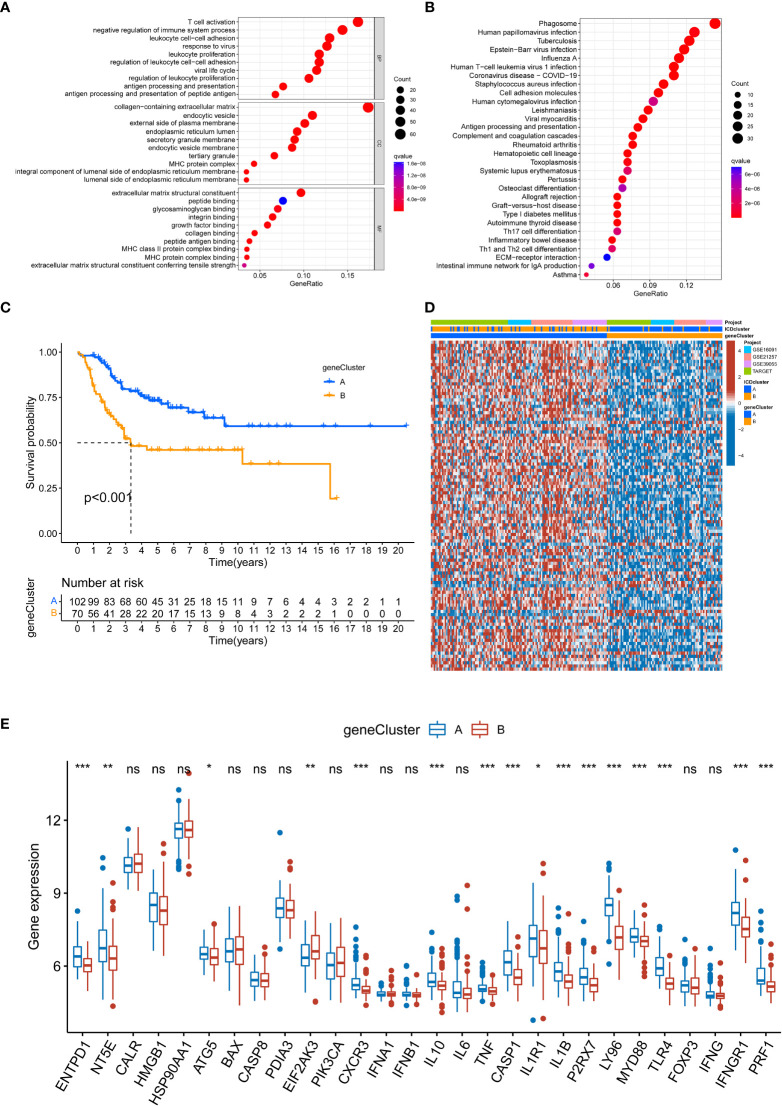
Establishment of gene classification based on ICD subtype-related DEGs. **(A, B)** GO and KEGG enrichment analyses based on DEGs between distinct ICD-related subtypes. **(C)** K-M Survival analysis of two different gene clusters in the OS cohort. **(D)** The relationships between the two gene cluster and ICD-related subtypes. **(E)** The expression level of ICD-related genes in two gene clusters. ICD, Immunogenic cell death; GO, Gene Ontology; KEGG, Kyoto Encyclopedia of Genes and Genomes; DEGs, Differentially expressed genes; K-M, Kaplan-Meier. ns, no significance, *p < 0.05, **p < 0.01, ***p < 0.001.

To comprehensively understand the potential biological functions of ICD-related subtypes in OS, we identified 348 DEGs (FDR<0.05 and |log2FC|≥1) associated with ICD subtypes by differential analysis. The heatmap and volcano of these DEGs are present in **Figure S3**. Then, we performed a functional enrichment analysis based on DEG, whose results showed that these ICD subtype-related DEGs were significantly enriched in immune and cancer-related pathways (**Figures 4A, B**). It was suggested that ICD greatly affects the carcinogenesis and immune microenvironment regulation of OS. Additionally, we used a consistent clustering algorithm to classify OS tumor patients into two gene clusters (A and B) based on these ICD subtype-related DEGs (**Figure S4**), similar to the phenotypic clustering of ICD. Meanwhile, the K-M survival analysis results denoted that gene cluster A had significantly better long-term survival than gene cluster B (**Figure 4C**). As expected, ICD-related gene cluster A was associated with the ICD-related B subtype (**Figure 4D**). Moreover, there was a difference in some ICD-related genes between the two gene clusters (**Figure 4E**). Hence, these results further confirmed the reliability of the ICD-related subtype and implied a crucial role of ICD in OS.

### Establishment and validation of a novel signature based on the ICD-related phenotype

**Figure 5:**
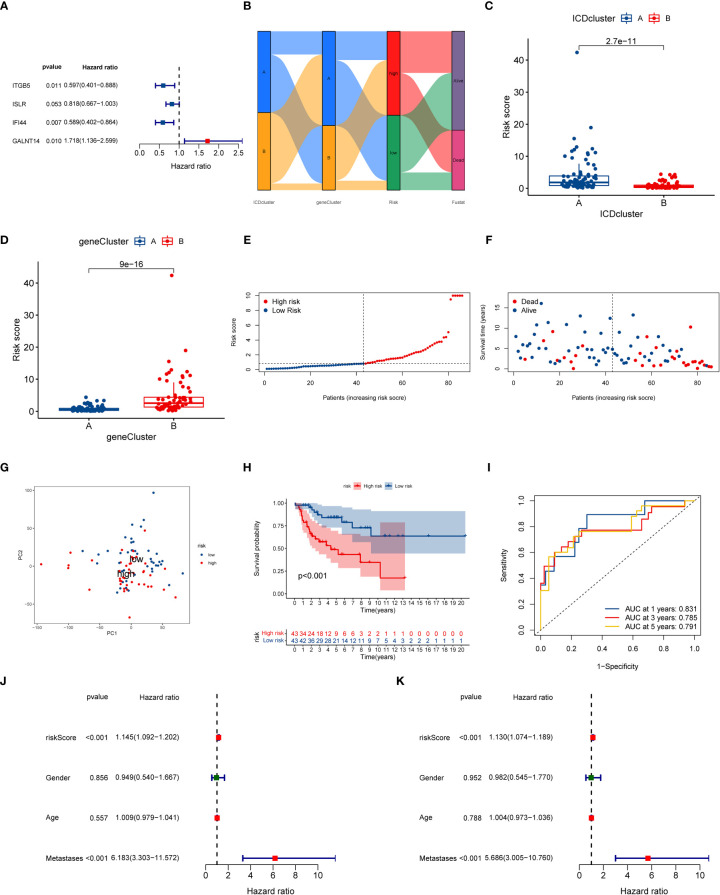
Construction of ICD-related signature in the training cohort. **(A)** Multivariate Cox regression analyses of the model gene. **(B)** Sankey plot of ICD-related subtype distribution in groups with different ICD risk scores and survival status. **(C)** The discrepancy in ICD risk score between the two ICD-related subtypes. **(D)** The discrepancy in ICD risk score between the two gene cluster. **(E)** The risk curve of each OS patient is reordered by risk score. **(F)** The scatter plot showed the overall survival status of each OS patient. **(G)** The PCA analysis is based on the ICD score in the training cohort. **(H)** K-M survival curve of overall survival by distinct ICD risk score groups for patients in the training cohort. **(I)** The ROC curves for predicting 1-, 3-, and 5-year OS survival according to ICD-related signature in the training cohort. **(J)** Univariate COX analysis result based on ICD risk score and clinical characteristics. **(K)** Multivariate COX analysis result based on ICD risk score and clinical characteristics. ICD, Immunogenic cell death; PCA, Principal component analysis; ROC, Receiver operating characteristic; OS, Osteosarcoma; K-M, Kaplan-Meier.

To understand the role of ICD subtypes in the prognostic assessment of OS patients, we establish a novel ICD-related signature according to ICD subtype-related DEGs. Initially, 103 subtype-related DEGs related to the prognosis were identified *via* univariate Cox regression analysis, which was used in subsequent analysis (**Table S5**). Subsequently, LASSO analysis was performed on 103 prognostic DEGs, and seven genes associated with survival prognosis were obtained according to the minimum partial likelihood of deviance (**Figure S5**). Next, those 7 DEGs were further subjected to multivariate COX regression analysis to construct an optimal prognostic signature according to the Akaike Information Criterion (AIC) value. As a result, a novel ICD-related signature including four candidate genes (ITGB5, ISLR, IFI44, and GALNT14) was successfully constructed. Among those candidate genes, ITGB5, ISLR, and IFI44 were low-risk genes, while GALNT14 was a high-risk gene (**Figure 5A** and **Table S6**). According to the multivariate COX regression analysis, the calculating method of the ICD risk scores equals (0.5412* expression of GALNT14) - (0.5158* expression of ITGB5) - (0.2011* expression of ISLR) - (0.5294* expression of IFI44). **Figure 5B** displays the population distribution for two ICD-related subtypes, two genotypes, and two ICD-related risk score groups. Inspiringly, the ICD subtype A had a higher ICD risk score than subtype B (**Figure 5C**), and the gene cluster B had a higher ICD risk score than gene cluster A (**Figure 5D**), suggesting that a lower ICD risk score may be strongly linked to the immune status (higher infiltrating immune cells) of OS.

At the same time, we further explored the relationship between ICD risk scores and survival status in OS. The distribution of ICD scores is shown in **Figures 5E, F**; as the ICD risk scores escalated, the death numbers rose, and the survival time decreased. The PCA analysis indicated a clear trend of separation between the ICD-related high and low-risk groups (**Figure 5G**). Moreover, K–M survival curves indicated that OS patients with high ICD risk scores significantly reduced the overall survival rate (**Figure 5H**). Then, we assessed the diagnostic value of the novel signature by using ROC curves. The results demonstrated that the AUCs at one year, three years, and five years were 0.831, 0.785, and 0.791, respectively, implying that the novel ICD-related signature has an excellent diagnostic value for the prognostic evaluation of OS (**Figure 5I**).

To further determine the predictive performance of the ICD-related signature, we performed validation analysis by using the test set and the entire test. **Figure S6** and **Figure S7** present the distribution of ICD risk scores, survival curves, and ROC curves according to the training set and the entire test. The validation results were consistent with the analytical results on the training set, intimating that the ICD-related signature has an excellent ability for prognosis prediction in OS.

### Independent prognostic value of the novel prognostic signature

Next, we further conducted a subgroup survival analysis to determine the applicability of this ICD-related signature. The results demonstrated that OS patients in the low-risk group had longer overall survival than that in the high-risk group regardless of the clinical subgroups, except for the metastases subgroup, implying the robustness of the prognostic prediction of our novel signature (**Figure S8**). More importantly, we also performed univariate and multivariate Cox analysis to explore whether the ICD risk score could predict the overall survival of OS independently. As illustrated in **Figures 5J, K**, the univariate Cox analysis shows that the risk score of ICD- related signature was associated with the prognosis of OS patients (HR = 1.145, 95% CI = 1.09-1.202, P < 0.001). Also, multivariate Cox analysis generate similar results (HR = 1.130, 95% CI = 1.074-1.198 P < 0.001). Hence, these results indicated that this ICD-related signature could be regarded as an independent risk factor for the prognosis prediction of the OS.

### Immunity statue in distinct ICD risk score groups

**Figure 6:**
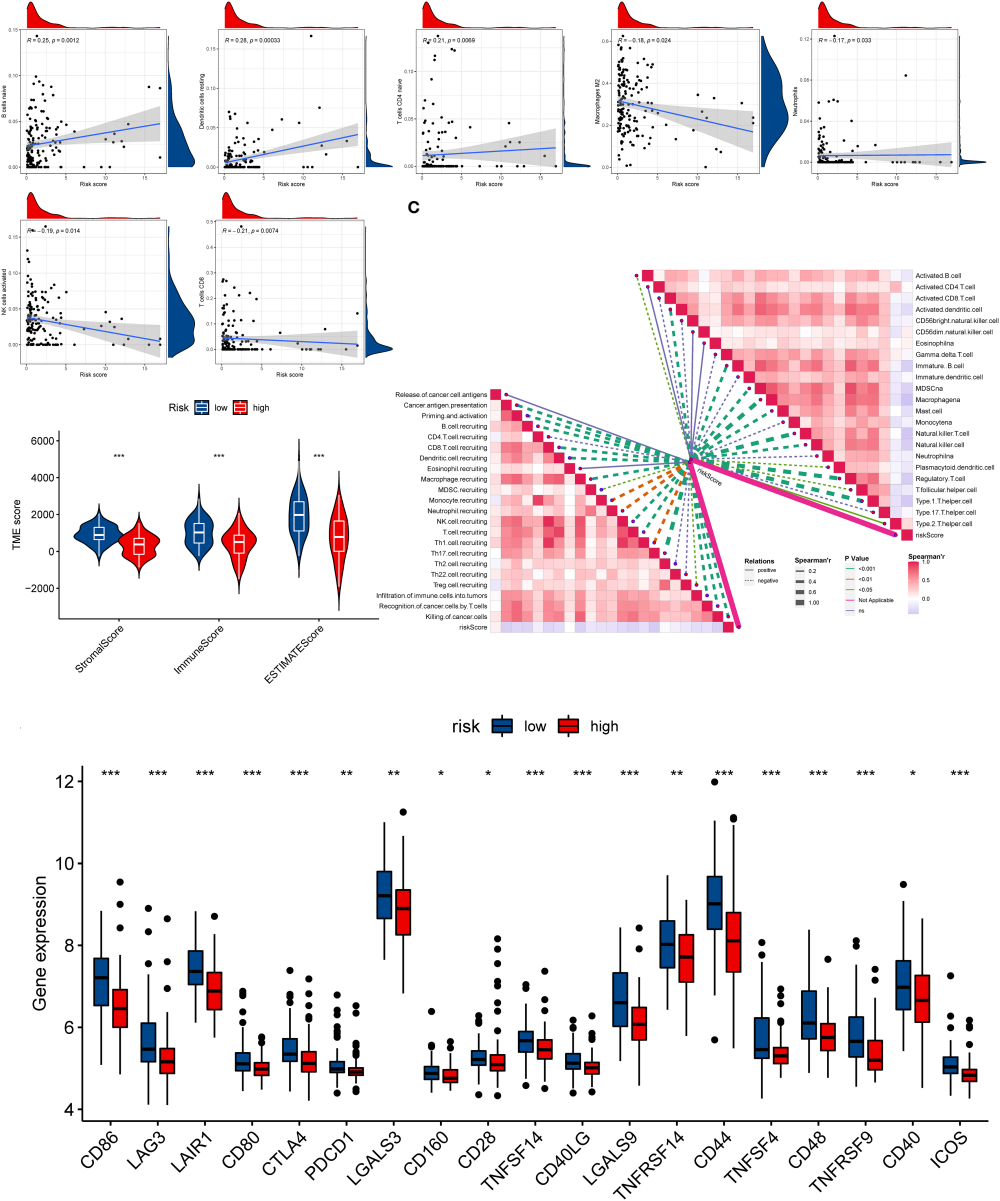
The evaluation of the immunity statute and checkpoints between the two distinct ICD risk groups. **(A)** The association between ICD-related signature and immune cell infiltration. **(B)** The difference in TME score between the two ICD risk groups. **(C)** Correlation of ICD risk score with immune cells and immune functions in OS. **(D)** The comparison of immune checkpoint expression between the low- and high-risk group. ICD, Immunogenic cell death; OS, Osteosarcoma; TME, tumor microenvironment. *p < 0.05, **p < 0.01, ***p < 0.001.

Similarly, the GSVA results revealed that the OS patients in low-risk group participated in some immune-related pathways (**Figure S9**), indicating a close relationship between the ICD-related signature and immunity of the OS patients. Consequently, the relationship between the ICD risk score and immunity was further examined in OS. Firstly, we explored the association between the ICD risk score and the extent of immune cell infiltration by utilizing the CIBERSORT algorithm. The results indicated that the ICD risk score was positively correlated with B cells naive, dendritic cells resting, and T cells CD4 naive, while negatively associated with macrophages M2, neutrophils, NK cells activated, and T cells CD8 (**Figure 6A**). Also, there was a significant association between some immune cells and these model genes (**Figure S10**). The ESTIMATE analysis result demonstrated that the TME score of the OS cohort in the low-risk group was higher than that in the high-risk group (**Figure 6B**). Furthermore, we also evaluated the association between ICD risk score and immune cells and immune-related function. We discovered that most immune cells and functions were inversely related to the ICD risk score (**Figure 6C**). Ultimately, we explored the connection between immune checkpoints and ICD-related signatures, and we observed that 19 immune checkpoints were differentially expressed in the two risk groups (**Figure 6D**). Collectively, these findings revealed that a low ICD score is associated with immune activation, and a better immune status may be helpful for the prognosis of OS.

### Drug susceptibility analysis

**Figure 7:**
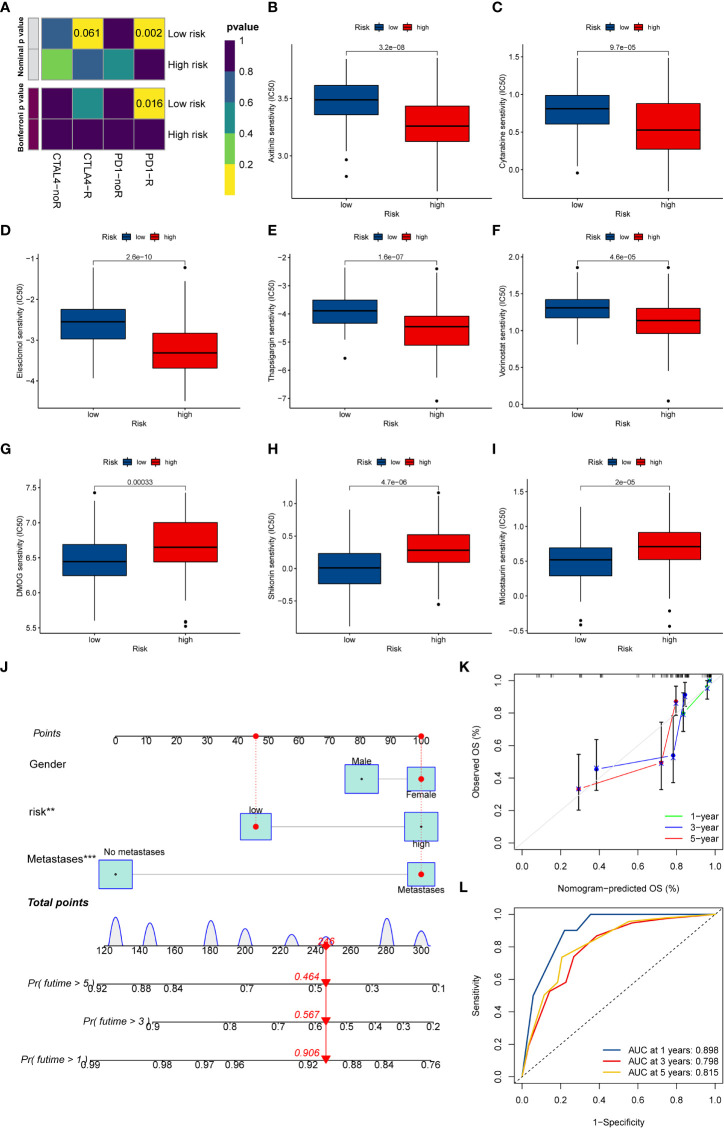
The potential clinical application of the novel signature. **(A)** Sensitivity prediction of OS patients with different ICD risk scores to the two immune checkpoint inhibitors. noR represent No response, and R represents the response. **(B–I)** Relationships between ICD risk score and chemotherapeutic sensitivity. **(J)** Nomogram based on the ICD risk score for predicting 1-, 3-, and 5-year survival rates of OS. **(K)** Calibration curves of the nomogram for predicting 1-, 3-, and 5-year survival rates. **(L)** ROC curves for predicting the 1-, 3-, and 5-year nomogram. ICD, Immunogenic cell death; OS, Osteosarcoma; ROC, Receiver operating characteristic. **p < 0.01, ***p < 0.001.

At present, anti-PD1/PD-L1 therapy plays a vital role in tumor immunotherapy. To further clarify the association between ICD-related signature and immunotherapy efficacy, we compared patient responses to immunotherapy and chemotherapy for the risk group by using subclass mapping (Submap). As displayed in **Figure 7A**, the OS patients in the low-risk group were more likely to respond to anti-PD1 therapy, which may be helpful for future investigation. Additionally, the chemotherapy agents were selected to compare the common drug sensitivity between patients with low- or high-risk populations. Additionally, we further selected chemotherapy agents to compare the common drug sensitivity between patients with low- or high-risk populations. Encouragingly, the OS populations in the low-risk group had lower IC50 of DMOG, midostaurin, and shikonin. In comparison, the IC50 for axitinib, cytarabine, elesclomol, thapsigargin, and vorinostat was higher than that in the high-risk group (**Figures 7B–I**). Collectively, these results denoted that the ICD-related signature may be used to guide future treatment for OS.

### Development of the nomogram

To better predict the prognosis of OS patients according to ICD-related signatures, we established a nomogram to expect the 1-, 3-, and 5-year survival rates of OS (**Figure 7J**). The clinical characteristics, including gender, metastases status, and risk scores, were enrolled in this nomogram. The results demonstrated that the survival rates decreased with increasing ICD scores. Subsequently, the calibration curve verified the accuracy of assessing the prediction nomogram. The results revealed that the survival rates predicted by the nomogram closely corresponded with actual survival outcomes, confirming the reliability of the nomogram (**Figure 7K**). In addition, the 1-, 3-, and 5-year AUC values of the nomogram were 0.898, 0.798, and 0.815, respectively (**Figure 7L**). Overall, the above results validated the firmness of this nomogram.

### These ICD-related signature genes in OS

**Figure 8:**
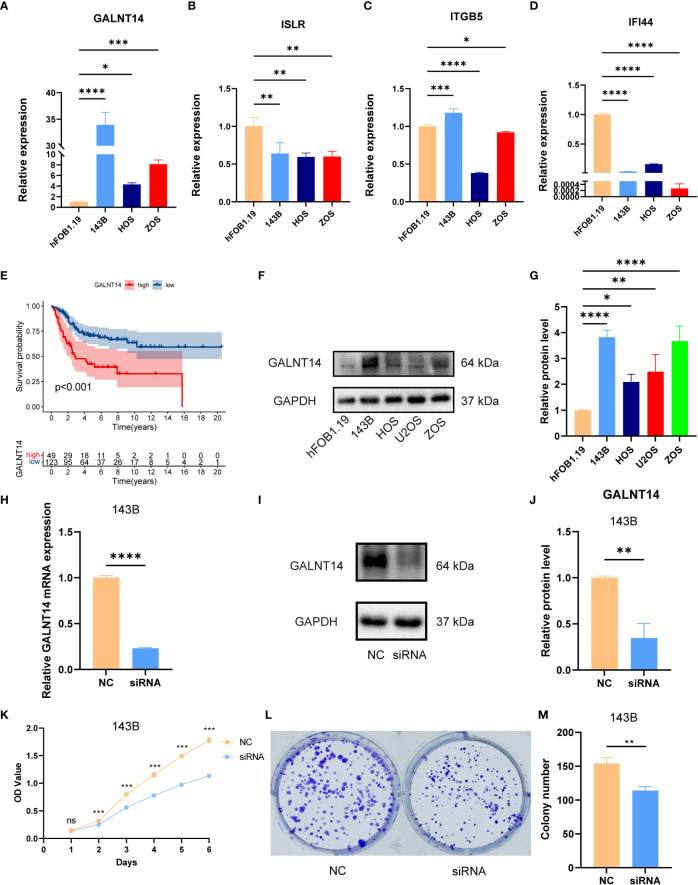
The expression of signature genes in OS and the effects of GALNT14 in OS. **(A–D)** The mRNA expression of GALNT14, ISLR, ITTGB5, and IFI44 was examined by RT-qPCR in hFOB 1.19 and osteosarcoma cell lines. **(E)** Kaplan–Meier curve of the expression level of GALNT14 on OS. **(F)** The protein expression of GALNT14 was measured by western blot in hFOB 1.19 and osteosarcoma cell lines. **(G)** The protein level of GALNT14 was quantified by the Image J software. **(H)** Knockdown of GALNT14 with siRNA in 143B cells was confirmed by RT-qPCR. **(I)** Knockdown of GALNT14 with siRNA in 143B cells was confirmed by western blot. **(J)** The protein level of GALNT14 was quantified by the Image J software. **(K)** Growth curves of cells were examined by CCK-8 assays after the knockdown of GALNT14 in 143B cells. **(L)** Colony formation assay was detected after the knockdown of GALNT14 in 143B cells. **(M)** Knockdown of GALNT14 inhibited proliferation of osteosarcoma cells 143B according to colony formation assay. ns, no significance,*p < 0.05, **p < 0.01, ***p < 0.001, ****p < 0.0001.

As shown in **Figure S11**, we observed that GALNT14 was overexpressed in high-risk groups, while ITGB5, ISLR, and IFI44 were diminished in the high-risk groups. Also, the RT-qPCR exhibited that GALNT14 was elevated, while ISLR, IFI44, and ITGB5(except for 143B) were downregulated in OS cell lines compared to that in normal cell line (**Figures 8A–D**). The RT-qPCR results were basically consistent with our previous bioinformatics analysis, which indirectly demonstrated the reliability of our signature.

Subsequently, we found that GALNT14 was correlated with an improved prognosis (**Figure 8E**), while ITGB5, ISLR, and IFI44 positively the survival rate (**Figure S11**). Since GALNT14 was the most and only important factor in the novel signature for predicting poor prognosis, we further explored the function of GALNT14 in OS. Consistently, the WB results revealed that the GALNT14 was significantly overexpressed in OS cell line (**Figures 8F, G**). To further evaluated the role of GALNT14 in OS, we knocked down the expression in the OS cell line through siRNA-GALNT14, the knockdown effect is shown in **Figures 8H–J**. Next, we utilized CCK-8 and clone formation assay in 143B with/without GALNT14 knockdown to investigate the impact of GALNT14 on proliferation in OS. With the downregulation of GALNT14 in 143B cells, the cell proliferation ability of 143B cells was greatly restrained (**Figures 8K–M**). In summary, these results revealed that the overexpression of GALNT14 is closely related to poor prognosis and malignant progress of OS.

## Discussion

OS is the most frequent primary malignancy of bone, with a high mortality rate in the predisposing population because of the recurrence and metastasis. (25). The major factors leading to the recurrence and metastasis of OS were the resistance to chemotherapy (26). The standard treatment for OS is comprehensive treatment such as neoadjuvant chemotherapy and surgery, etc. (27, 28). However, the prognosis of OS with metastasis or recurrent recurrence has not been greatly improved in the last decades, and the course of treatment has reached a frustrating plateau (29, 30).

Although significant advances in immunotherapy have been achieved in cancer areas, the effect on osteosarcoma has not been encouraging (31). Therefore, developing a method to judge or enhance the immunotherapy response and increase the chemotherapeutic drug sensitivity demonstrates great prospects in improving the prognosis of OS. ICD is a unique regulatory cell death, which can trigger an antigen-specific adaptive immune response through danger signals or DAMPs generation (32, 33). A growing body of studies has proved that ICD is an important predictor of effective antitumor immunity, which holds substantial promise for the treatment strategies of malignant tumors (11, 16, 34). Although the relationship between ICD and osteosarcoma has been previously explored by Jiaqi Yang et al. (35), the correlation between OS and ICD is challenging to grasp. Different to previous studies, we not only systematically inveterated the ICD-related genes in OS phenotyping and TME from the bioinformatics level but also further verified the role of ICD prognostic genes in OS by *in vitro* experiments. In addition, there are many other characteristics in our study, such as signature construction methods and corresponding results. The signature constructed by Jiaqi Yang et al. was composed of BAMBI, TMCC2, NOX4, DKK1, and CBS. Yet, we identified GALNT14, ITGB5, ISLR, and IFI44 as the prognostic gene and verified that GALNT14 is closely associated with poor prognosis and malignant progress of OS.

The previous studies confirmed that the prognostic risk signatures based on the subtype-associated DEGs are an excellent tool applicable to the prognosis prediction of individual tumor patients (36–38). For example, Wei Song et al. identified a signature based on the pyroptosis subtype DEGs, which paved a new path for prognosis prediction in colorectal cancer (37). Similarly, we have constructed a prognostic risk signature consisting of four ICD subtypes related to DEGs. As predicted, the B subtype group had a higher ICD risk score, and the OS patients with a low ICD risk score had better survival status. These results were confirmed again in the test and the whole cohort. On the other hand, the ROC curve, PCA analysis, and subgroup survival analysis further determine the excellent predictive performance of the ICD-related signature. Moreover, the univariate and multivariate Cox regression analysis results indicated that the ICD risk score was an independent prognostic factor for OS. Therefore, our findings denote that the novel ICD-related risk signature can be used as a stable predictor for OS, which could be helpful for patient stratification and prognosis prediction.

TME has been proven to play an important role in carcinogenesis, progression, and drug resistance (39–41). In this study, it was found that ICD-related B subtype was associated with immune activation and lower ICD risk scores. Then, the relevance of ICD risk scores with immune status was clarified using CIBERSORT, ESTIMATE, and ssGSEA algorithms. Consistent with previous analyses, the results of ESTIMATE and ssGSEA demonstrated that ICD risk score was positively associated with TME score and immune cell function, which further imply that prognostic differences in distinct ICD risk groups were related to immune activation. Similar results were reported by Ting Lei et al., who demonstrated that OS patients with low ferroptosis-related risk have a higher immune score, more active immune status, and better survival prognosis(LeiQian^42^). We also observed the differences in the percentage of immune cell infiltration between the two ICD risk groups, especially in B cells naive, dendritic cells quiescence, T cells CD4 naive, macrophages M2, neutrophils, NK cells activated, and T cells CD8. According to previous studies, B cells play a supportive role in tumor progression by stimulating angiogenesis, pro-inflammatory microenvironment, and inhibiting T cell activation, thereby affecting the clinical prognosis of tumors (43). On the other hand, CD8-positive T cells serve an essential role in suppressing tumor growth by recognizing tumor-associated antigens (44). Therefore, it is reasonable to believe that a better tumor immune microenvironment and immune cell infiltration may contribute to improving the survival prognosis of OS, which could help provide new insights into the improvement of immunotherapy for OS.

Recently, immune checkpoint blockade therapy has become the most promising immunotherapy, and the reaction to immune checkpoint inhibitors is a critical feature of satisfactory treatment (45, 46). Interestingly, most immune checkpoints, including CTLA4, were significantly different between different ICD risk score groups. In addition, the patients with low ICD risk scores are better responsive to anti-PD1 therapy. Indeed, the approach to immunotherapy response prediction is widely recognized. For example, Chengcheng Shi et al. reported that the OS cohort with a low unfolded protein response risk score was more sensitive to anti-PD1 therapy (47). These results demonstrate that the novel ICD-related risk signature acts as a potential marker to evaluate immunotherapy response in the OS. In addition, the chemosensitivity to candidate anticancer drugs in distinct ICD risk groups was compared. The OS cohorts in the high-risk group were more sensitive to axitinib, cytarabine, elesclomol, thapsigargin, and vorinostat. In contrast, the low ICD-risk patients displayed better responses to DMOG, Midostaurin, and Shikonin. Pettke A et al. reported that vorinostatin synergistically enhanced the cytotoxicity of doxorubicin and cisplatin in OS, which may be a promising addition to present treatment regimens (48). Midostaurin (PKC412), a derivative of staurosporine, has been proven to induce the apoptosis of Ewing’s sarcoma cell lines and significantly suppress xenograft tumor growth (49). In brief, these drugs or combination therapies could improve therapeutic efficiency, and the novel ICD-related signature could facilitate accurate customization of the OS treatment regimens.

Ultimately, the expression of these genes consisting of the novel ICD-related signature by *in vitro* experiments was verified. Encouragingly, our result demonstrated that GALNT14 was elevated in OS as the prognostic risk factor. On the other hand, the expression of ITGB5, ISLR, and IFI44 was down-regulated in OS, which verified that they were protective prognostic factors for OS. These signature genes have attracted widespread attention in the cancer field. Accumulating evidence has shown that GALNT14 was over-expressed in tumors such as breast, lung, and ovarian cancer to promote the process of tumor malignancy (50–52). Recently, several studies have shown associations between IFI44 and tumors (53–55). For example, L.C. Hallen et al. reported that IFI44 inhibits melanoma cell proliferation and abrogates ERK signalling through intracellular binding GTP (53). Therefore, these results further verified the veracity of our analysis, which will provide new insights into future studies of biomarkers for OS.

It is worth noting that there are still several inevitable limitations that should be improved in the future. First, although we verified the ICD-related signature using different cohorts and *in vitro* experiments, the real tumor tissue RNA- seq datasets to further verify our results in the future are also important. Collecting more clinical cohorts could help reassert the value of ICD-related signatures, which would be in our plans. In addition, we just preliminarily proved that GALNT14 could promote the proliferation of OS cells, while the specific mechanism of its regulation of the proliferation of OS cells and whether it is related to protein glycosylation remains requires further biomedical experiments. Nevertheless, the current results are encouraging and noteworthy in the field of prognosis prediction, personalized and accurate immunotherapy strategies for OS.

## Conclusions

In summary, we systematically analyzed the promising functions of ICD-related genes to prognosis, TME, immune response, and chemotherapeutic drug sensitivity and verified the prognostic role of ICD-related genes *in vitro* experiments, providing guidance for personalized and accurate immunotherapy strategies for OS.

## Data availability statement

Publicly available datasets were analyzed in this study. This data can be found here: https://www.ncbi.nlm.nih.gov/geo/, GSE16091, GSE21257.

## Author contributions

SH and CT contributed to the conception and made final approval of the version, ZYL and BL performed the study concept and design and wrote the manuscript. ZYL performed the experiment. CF, CL, HW, PL, HZ, and ZHL helped with data analysis. All authors contributed to the manuscript revision and read and approved the submitted version.

## Funding

This work was supported by the National Natural Foundation of China (82272664, 81902745), Hunan Provincial Natural Science Foundation of China (2022JJ30843), the Science and Technology Development Fund Guided by Central Government (2021Szvup169), Hunan Provincial Administration of Traditional Chinese Medicine Project (D2022117), the Scientific Research Program of Hunan Provincial Health Commission (B202304077077), and the Rehabilitation Project of Hunan Disabled Persons' Federation (2022XK0215).

## Conflict of interest

The authors declare that the research was conducted in the absence of any commercial or financial relationships that could be construed as a potential conflict of interest.

## Publisher’s note

All claims expressed in this article are solely those of the authors and do not necessarily represent those of their affiliated organizations, or those of the publisher, the editors and the reviewers. Any product that may be evaluated in this article, or claim that may be made by its manufacturer, is not guaranteed or endorsed by the publisher.

## Abbreviations

OS, Osteosarcoma; TARGET, Therapeutically Applicable Research to Generate Effective Treatments; ICD, Immunogenic Cell Death; DAMPs, Dying Tumor Cells Release Damage-Associated Molecular Patterns; CRT, Surface-Exposed Calreticulin; HMGB1, High Mobility Group Protein B1; APCs, Antigen-Presenting Cells; FPKM, Fragments Per Kilobase Per Million; TPM, Transcript To Millions Per Kilobase; GSVA, Gene Set Variation Analysis; ESTIMATE, Estimation of Stromal and Immune Cells In Malignant Tumor tissues using expression; ssGSEA, Single-Sample Gene Set Enrichment Analysis; PCA, Principal component analysis; TME, Tumor Microenvironment; DEG, Difference Expressed Gene; GO, Gene Ontology; KEGG, Kyoto Encyclopedia of Genes and Genomes; LASSO, Least Absolute Shrinkage and Selection Operator; K-M, Kaplan-Meier; ROC, Receiver Operating Characteristic; TIDE, Tumor Immune Dysfunction and Exclusion; IC50, Semi-Inhibitory Concentration; AIC, Akaike Information Criterion; Submap, Subclass Mapping; DCA, Decision Curve Analysis.
