# Supplementary figures and images for: Molecular characterization of immunogenic cell death indicates prognosis and tumor microenvironment infiltration in osteosarcoma
Source: Front Immunol. 2022 Dec 9;13:1071636. doi: 10.3389/fimmu.2022.1071636 (PMC9780438; doi:10.3389/fimmu.2022.1071636)

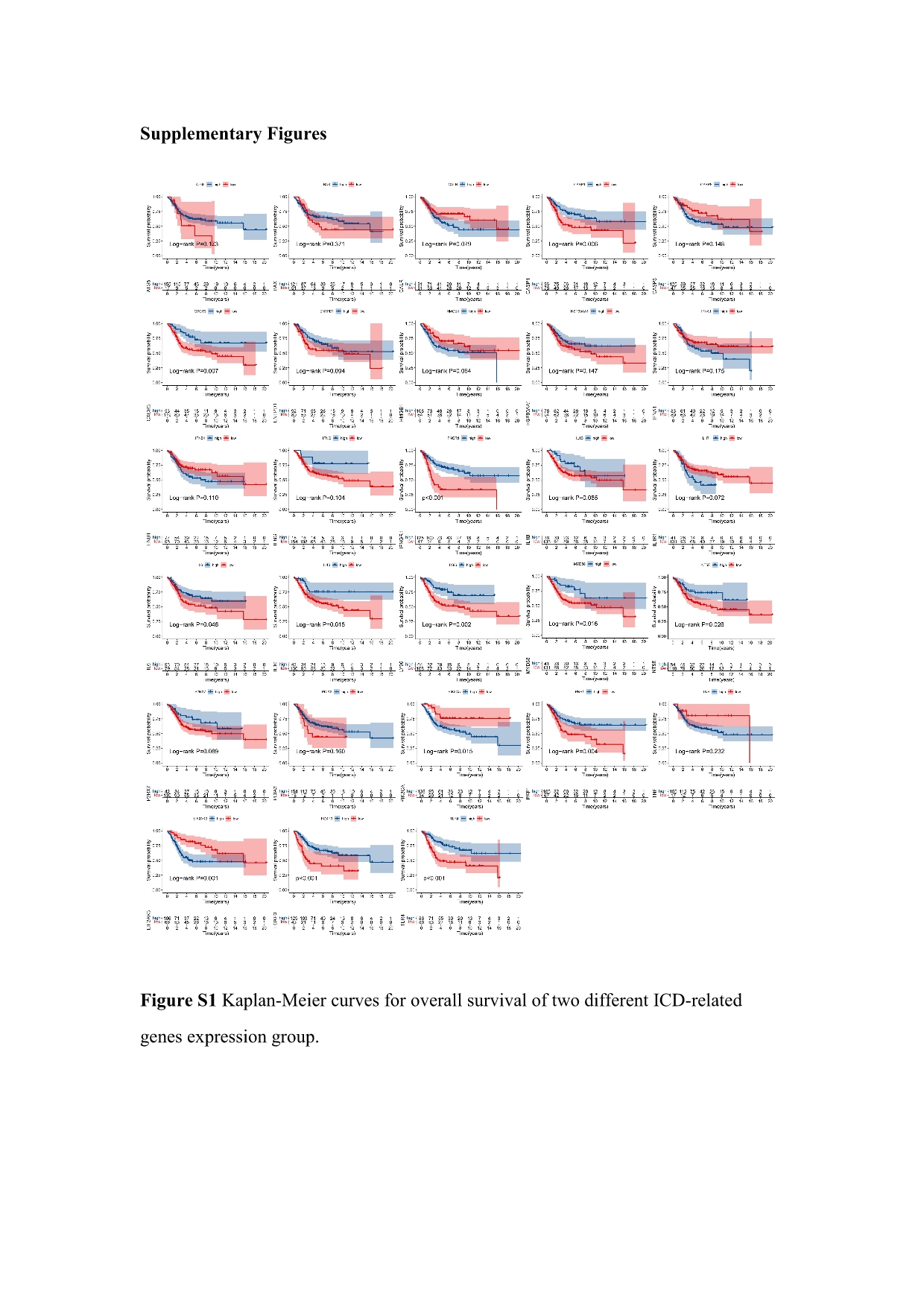

Supplement: Supplementary file 2 [file Image_1.jpeg]

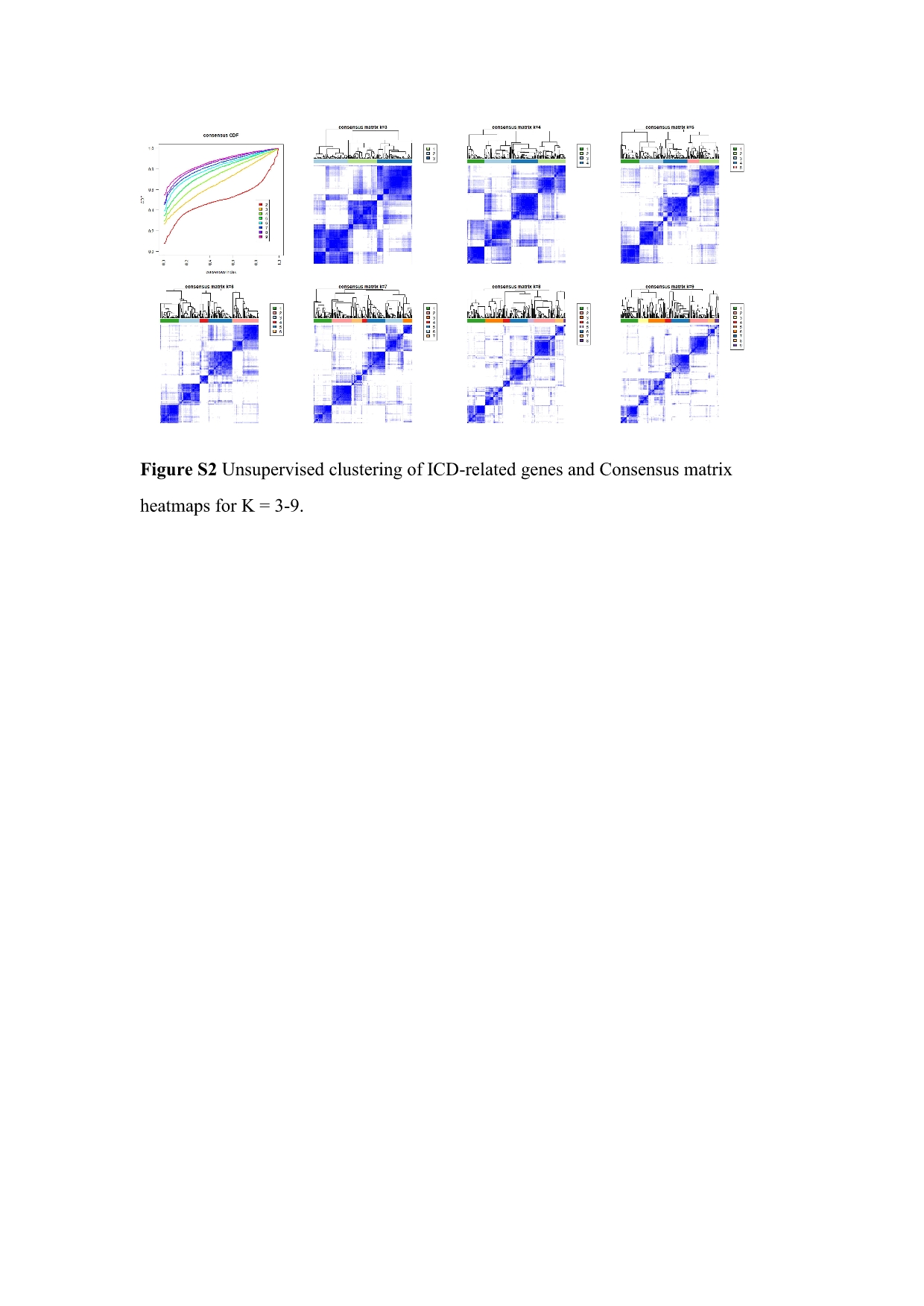

Supplement: Supplementary file 3 [file Image_2.jpeg]

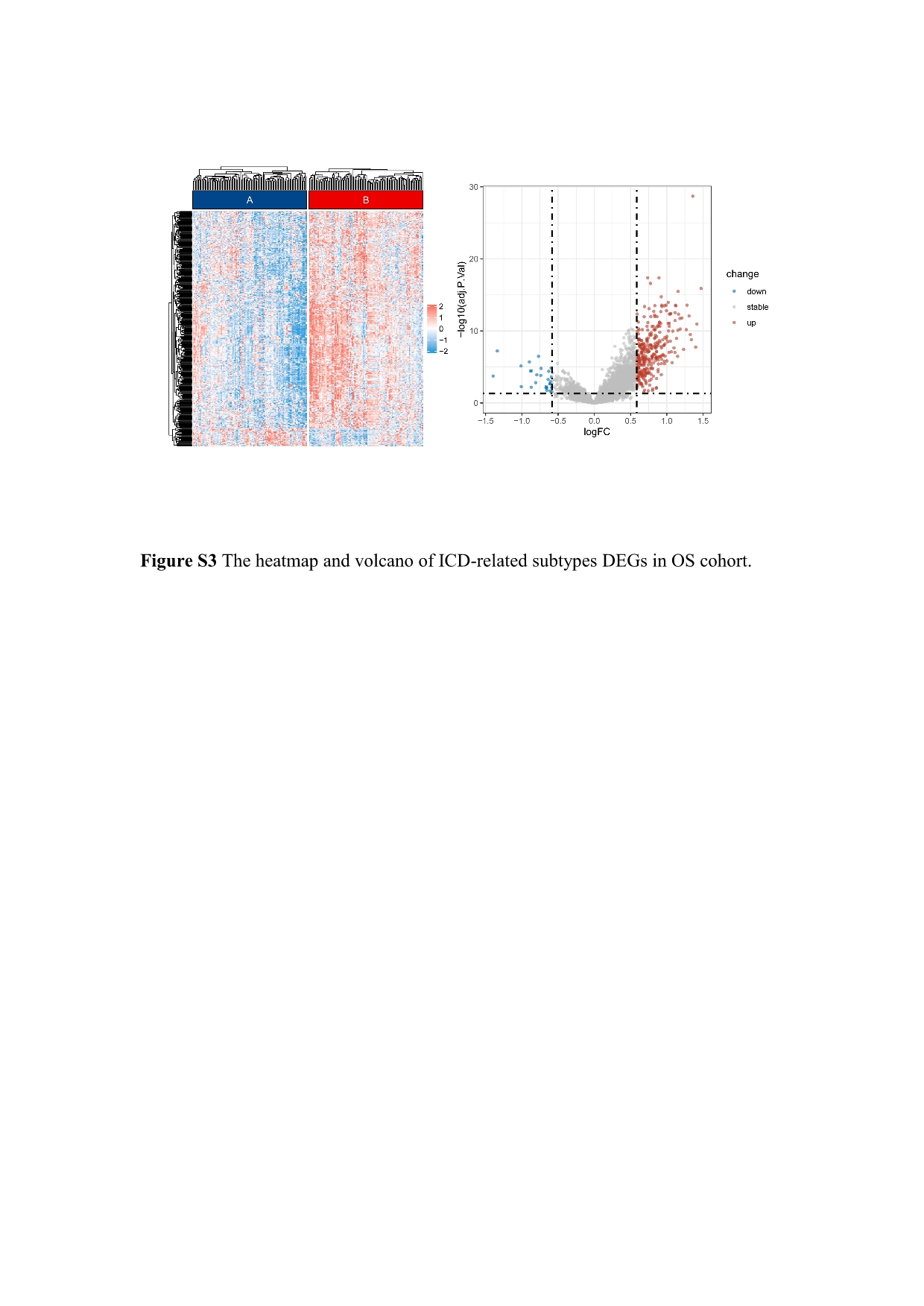

Supplement: Supplementary file 4 [file Image_3.jpeg]

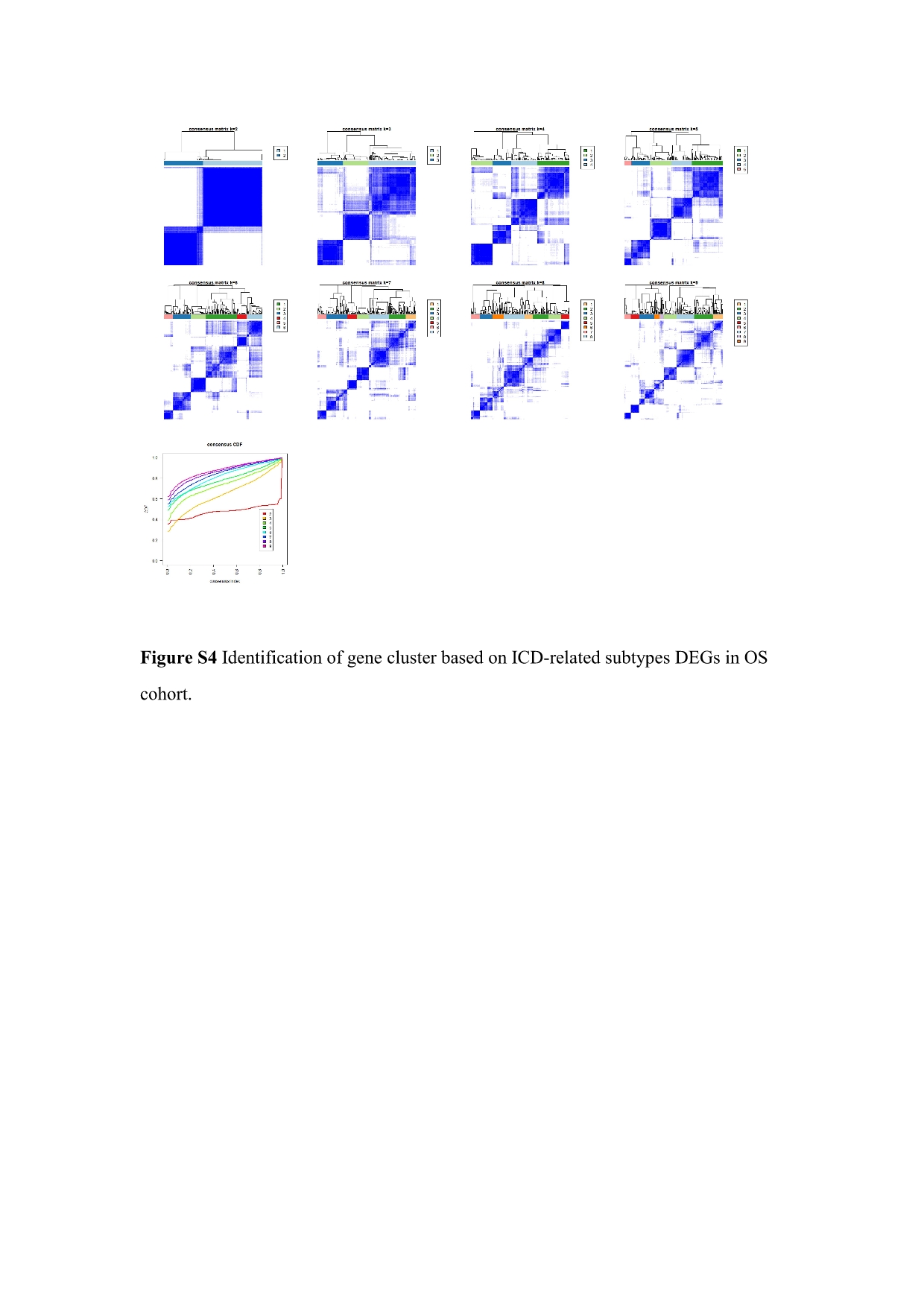

Supplement: Supplementary file 5 [file Image_4.jpeg]

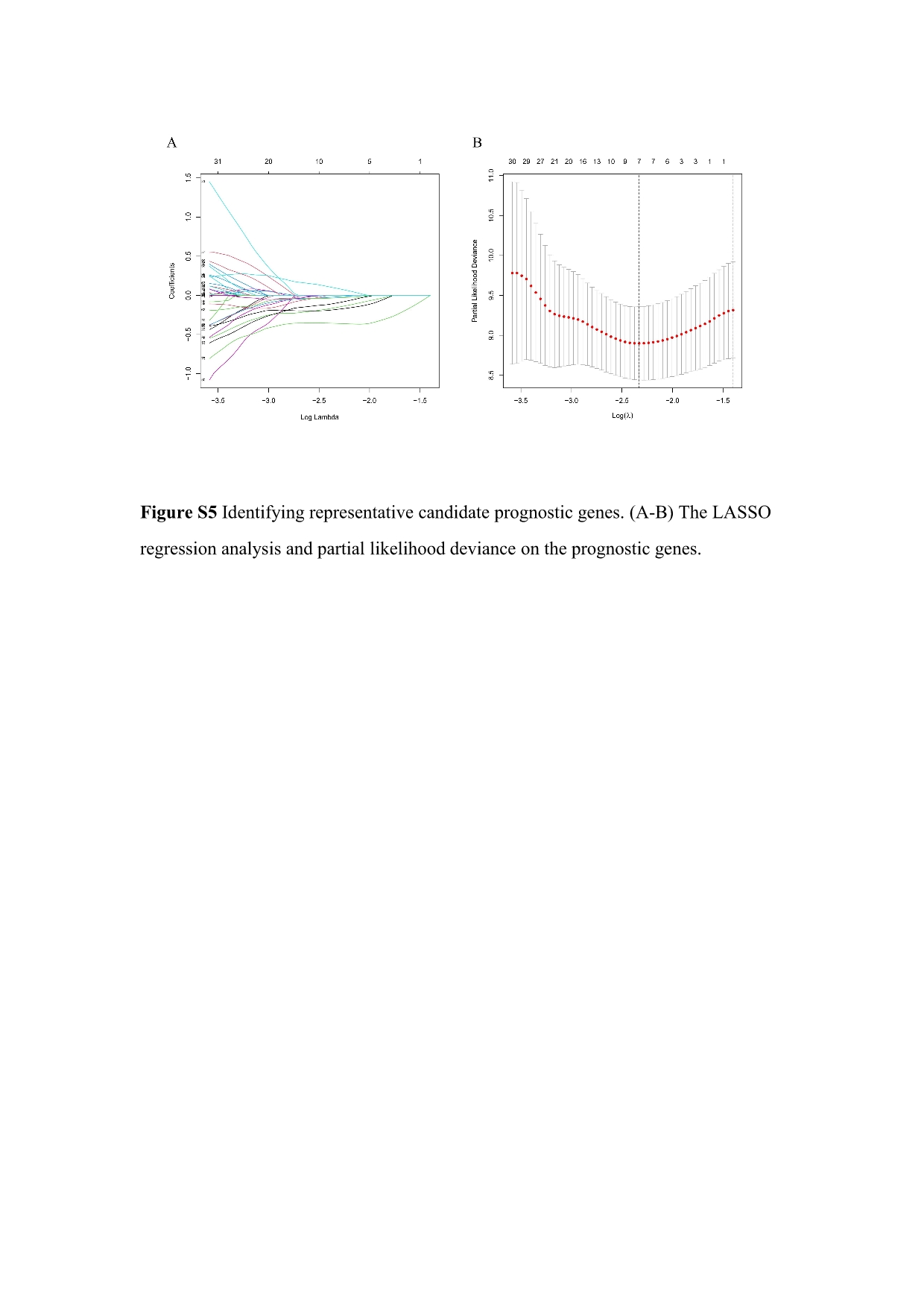

Supplement: Supplementary file 6 [file Image_5.jpeg]

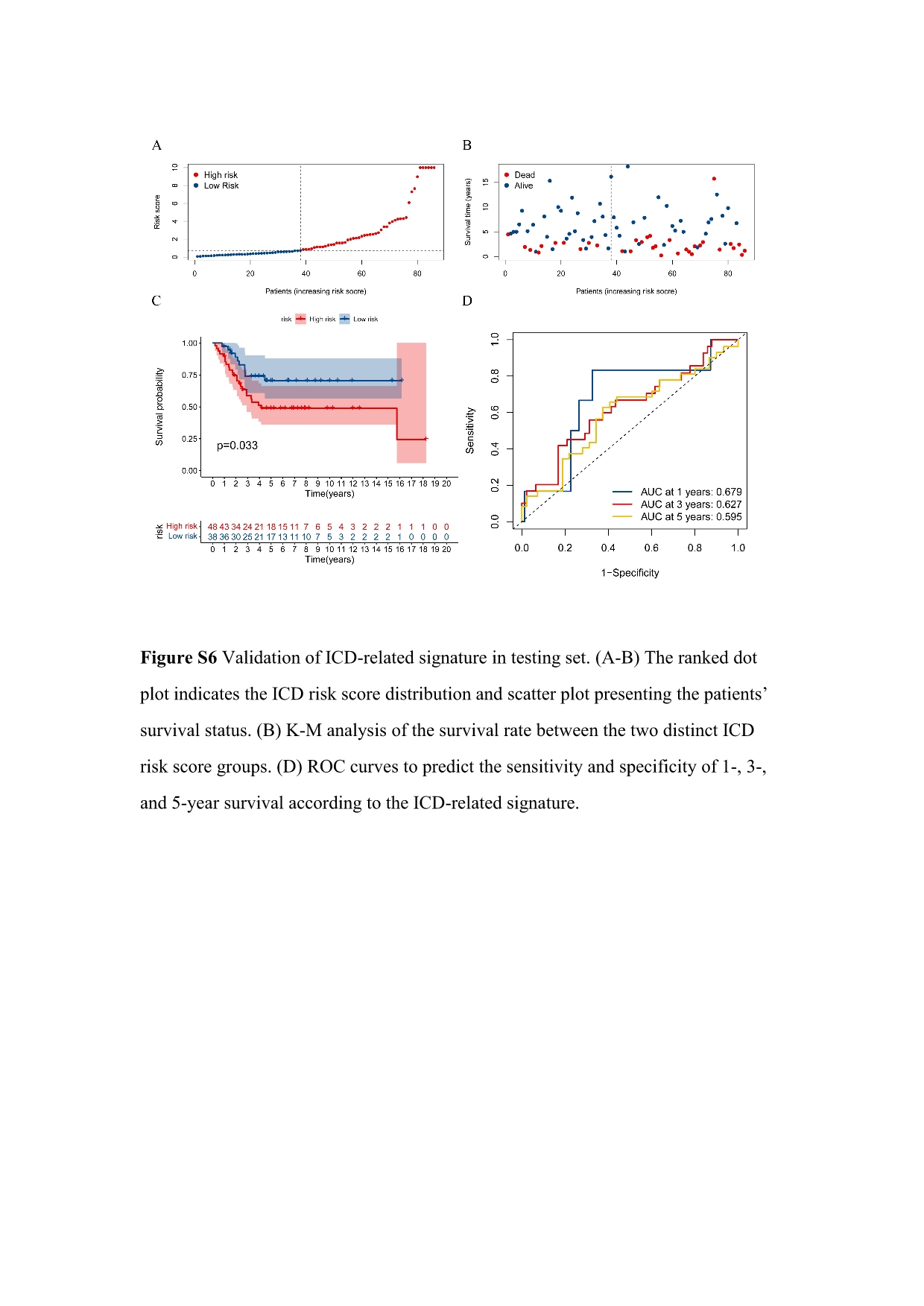

Supplement: Supplementary file 7 [file Image_6.jpeg]

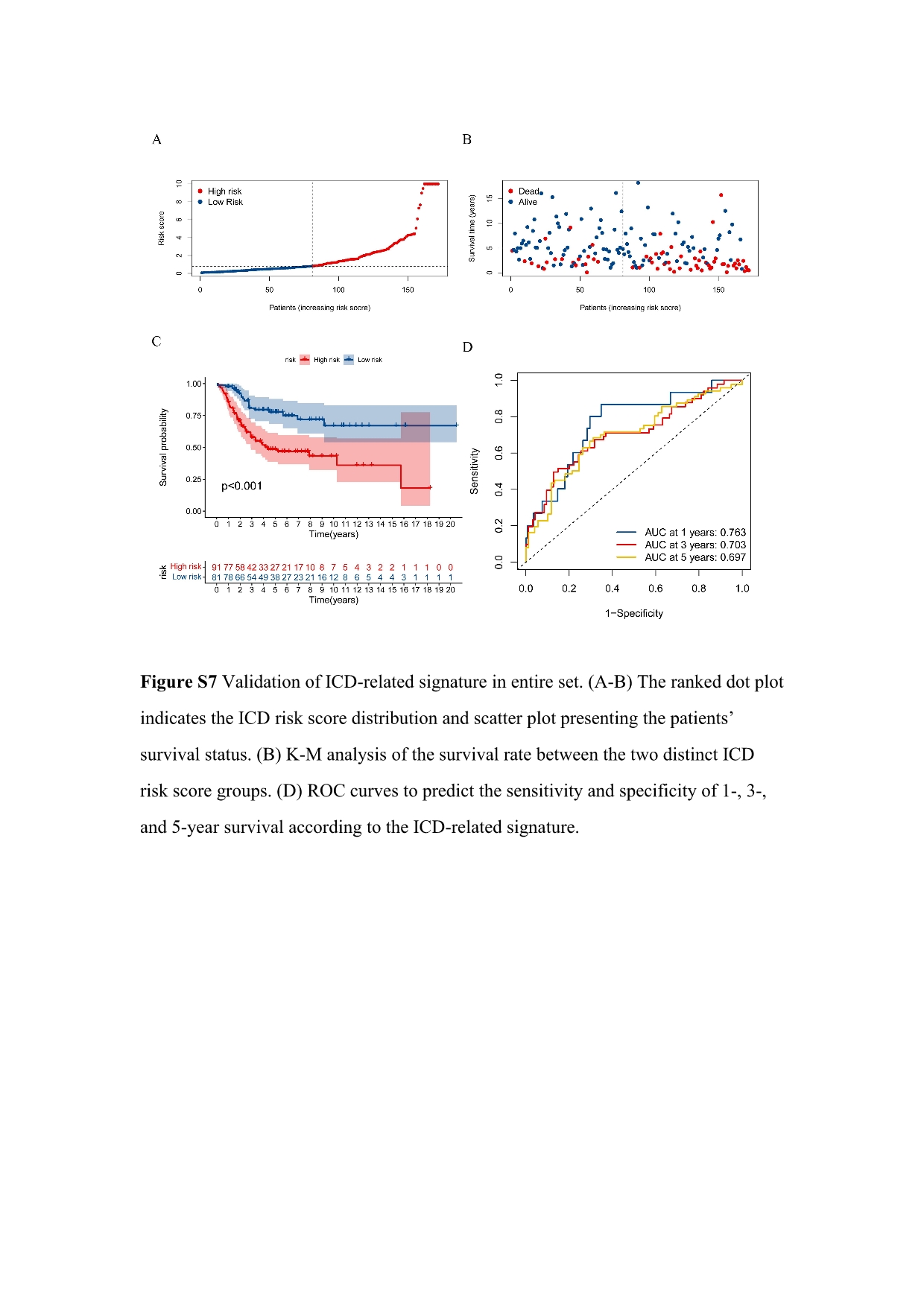

Supplement: Supplementary file 8 [file Image_7.jpeg]

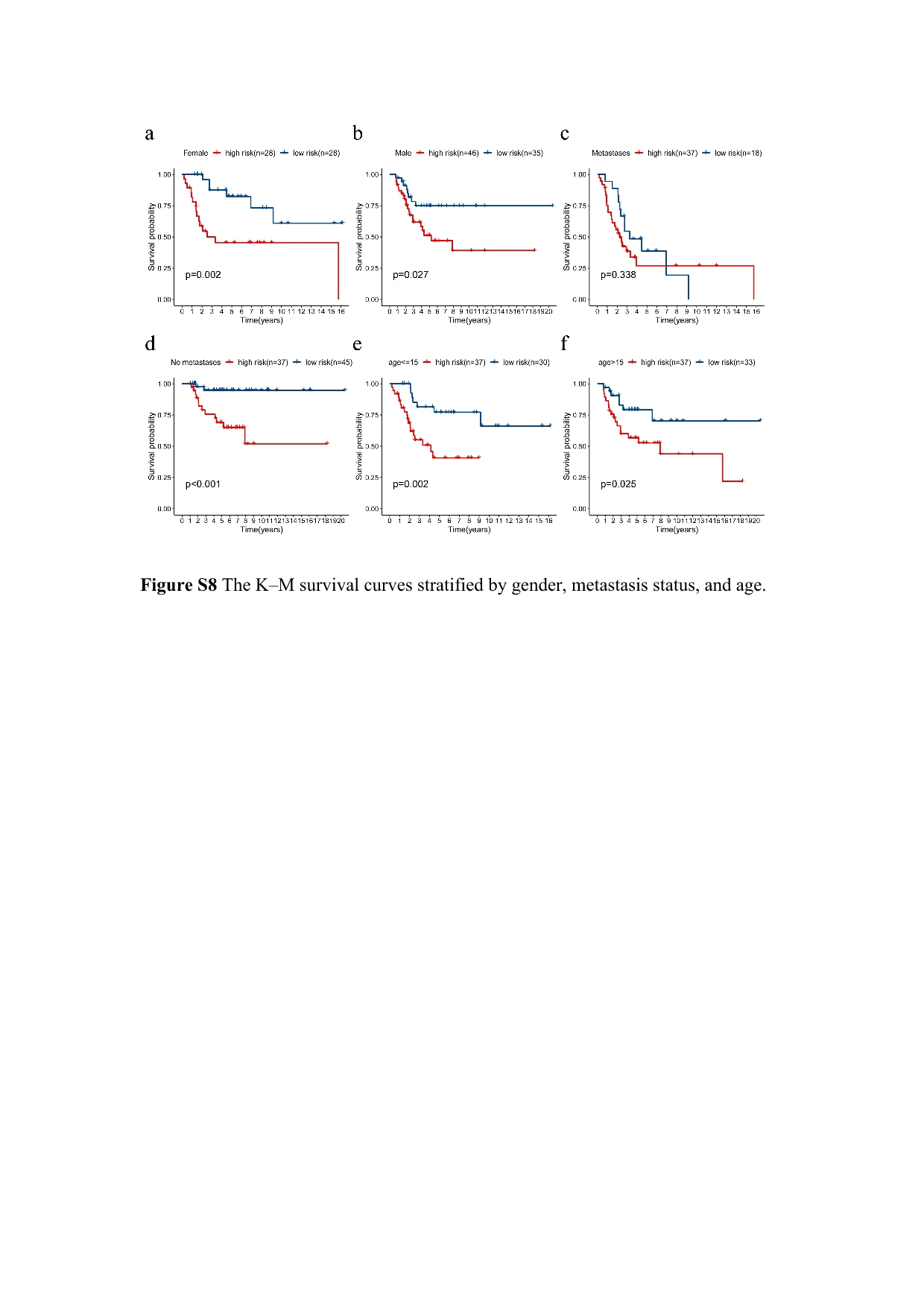

Supplement: Supplementary file 9 [file Image_8.jpeg]

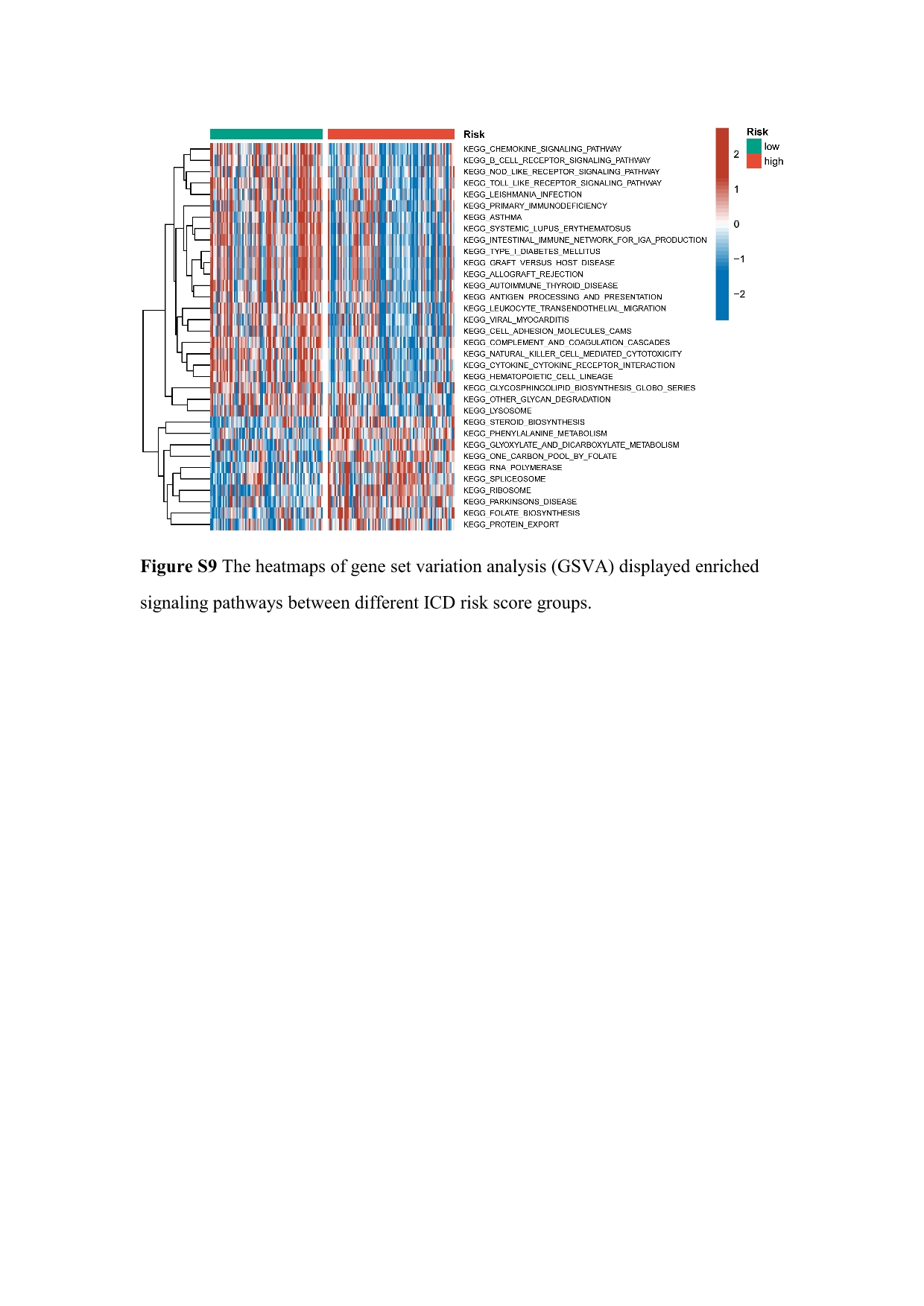

Supplement: Supplementary file 10 [file Image_9.jpeg]

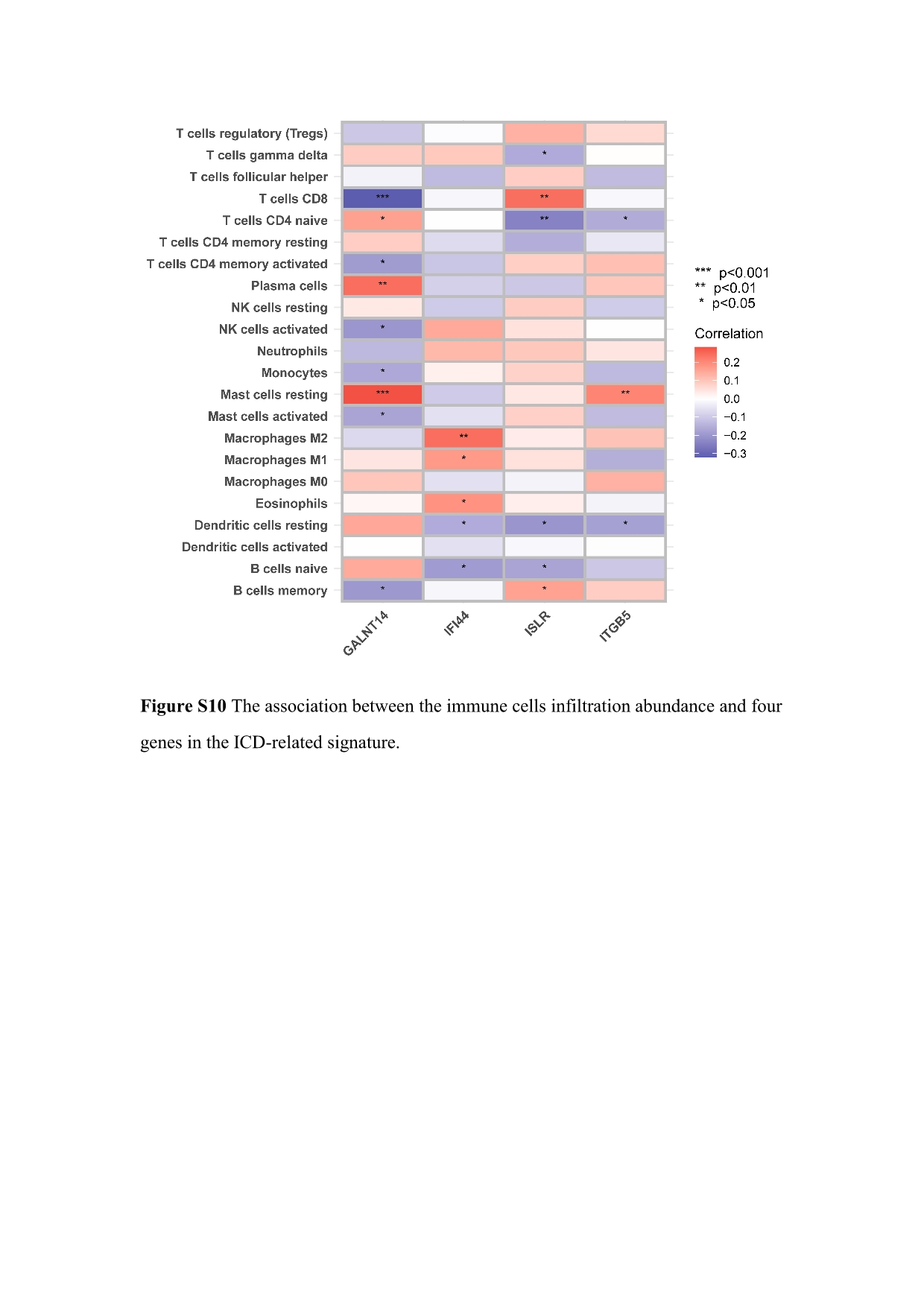

Supplement: Supplementary file 11 [file Image_10.jpeg]

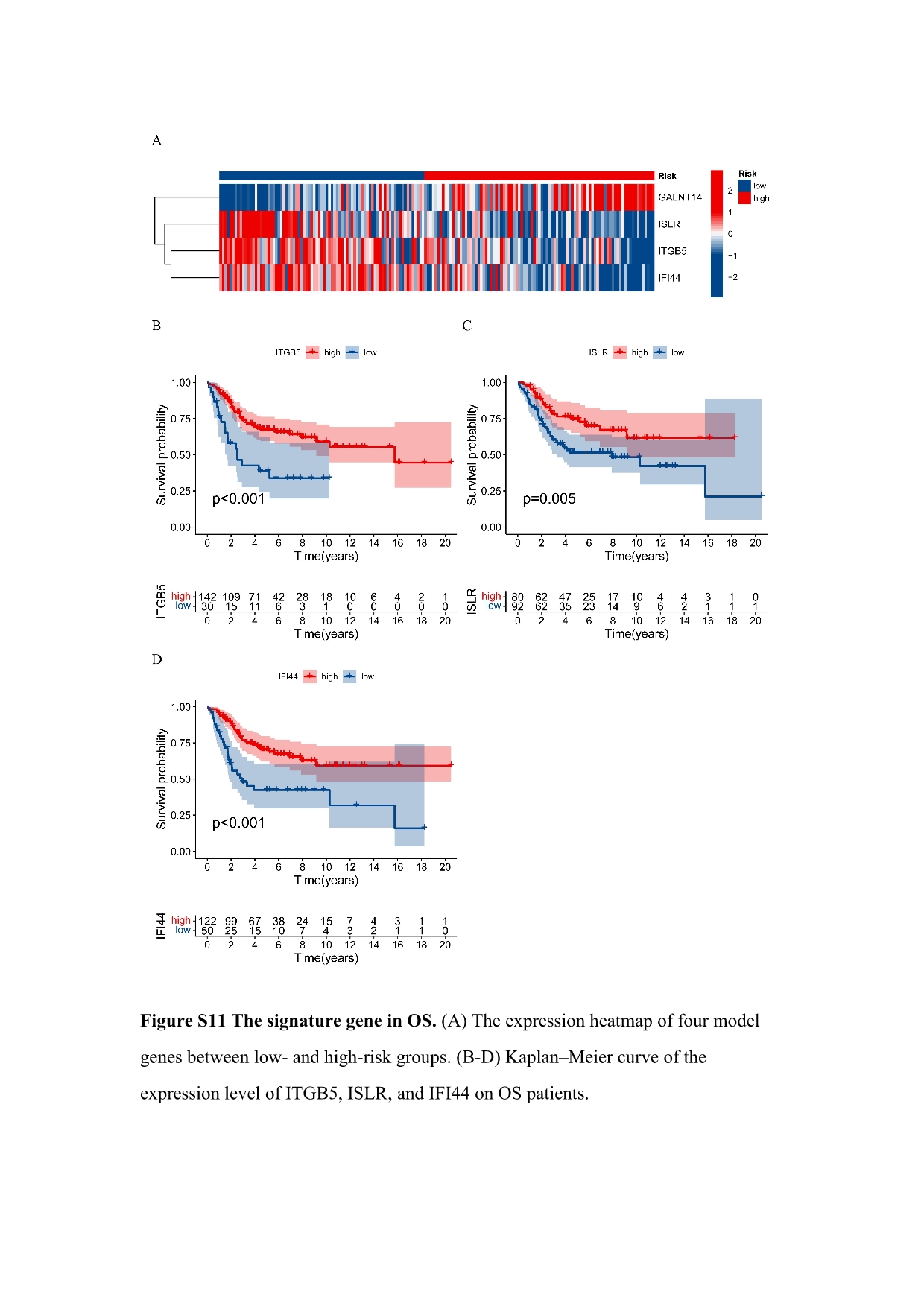

Supplement: Supplementary file 12 [file Image_11.jpeg]
